# A Study on Three-Dimensional Flexible Mesh Influence on the Stability of Reserved Tunnels in Cemented Backfill

**DOI:** 10.3390/ma18143291

**Published:** 2025-07-12

**Authors:** Xiaosheng Liu, Weijun Wang, Hao Li

**Affiliations:** 1 School of Resources, Environment and Safety Engineering, Hunan University of Science and Technology, Xiangtan 411201, China; 1010038@hnust.edu.cn (W.W.); 22020101044@mail.hnust.edu.cn (H.L.); 2 Work Safety Key Lab on Prevention and Control of Gas and Roof Disasters for Southern Goal Mines, Hunan University of Science and Technology, Xiangtan 411201, China

**Keywords:** three-dimensional flexible mesh, cemented backfill, mechanical characteristic, strengthening effect, numerical simulation

## Abstract

Ordinary backfill has characteristics such as low compressive strength, low tensile strength, and easy bending, which cannot meet the stability requirements of reserved tunnels, but three-dimensional flexible mesh can be added to improve it. In this paper, mechanical characteristics and displacement were taken as the evaluation index, an optimal three-dimensional flexible mesh was studied by a laboratory experiment of small samples, then backfill with a reserved roadway was used to carry out a large-sample similarity simulation experiment, and finally, a numerical simulation was carried out. The research shows that the three-dimensional flexible mesh had a strengthening effect on the backfill, especially on the tensile strength and shear strength of the backfill. The strengths increased by 1.57~2.00 times and 2.00~2.56 times, respectively. After backfill is damaged by external forces, three-dimensional flexible mesh can also hinder the detachment of backfill fragments. The effect of the three-dimensional flexible mesh on the backfill under static pressure was calculated by using numerical simulation, and it was found that the three-dimensional flexible mesh played an effective support role for the roadway inside the backfill, effectively reducing the displacement of the roadway roof by 21.43% and the strain energy by 40%.

## 1. Introduction

According to the concept of green mining, mines are gradually adopting filling mining methods to improve resource utilization ratio, reduce environmental pollution, and ensure safety issues [1,2,3]. However, when mining pillars or adjacent mining stopes, it is necessary to excavate tunnels in the backfill material [4,5]. Due to the lower strength and larger plastic zone of the backfill compared to the original rock, severe collapse has occurred during the excavation process, and the support strength was relatively high, causing serious difficulties for production [5,6]. Therefore, this article proposes a reserved roadway technology to reduce interference with the backfill, thereby reducing the damage caused to the backfill.

Reserved tunnel technology can reduce the damage to the backfill, but the backfill has the characteristics of low strength and large plastic deformation. When subjected to external forces, a creep phenomenon will occur, so, over time, the tunnel section will still decrease [6,7,8]. Therefore, it is necessary to strengthen tunnel support or improve the performance of backfill materials. At present, tunnel support generally includes anchor rods, shotcrete, and steel frame support [9,10]. However, these support methods have high requirements for the surrounding rock and support technology, and the cost is relatively large. Therefore, methods to improve the performance of the backfill can be utilized. In order to increase the compressive strength, tensile strength, and bending resistance of the backfill material and reduce deformation, some scholars have proposed the method of adding fibers [11,12,13]. Commonly used fibers include polypropylene fiber, straw fiber, glass fiber, etc. [14,15,16,17]. Xu Wenbin et al. conducted experiments on reinforcing polypropylene fibers and found that adding a certain quantity of fibers can increase the strength of the backfill material and constrain its deformation [15]. Wu Shenghai et al. also conducted relevant research and found that both polypropylene fibers and glass fibers can improve the strength of backfill, with polypropylene fibers having a more significant impact on tensile strength [16]. However, the fibers were discrete and prone to agglomeration, so the addition of fibers will lead to a decrease in the fluidity of the filling slurry, affecting its transportation. At the same time, it will also increase the difficulty of stirring, and the distribution of fibers will not be uniform enough. In order to avoid the occurrence of the above problems, this study proposes a method of adding three-dimensional flexible mesh to the backfill to improve its strength, which also refines the current body of knowledge. Some studies have shown that flexible mesh can effectively improve the strength of the backfill material [18].

Based on the foundation and shortcomings of the above research, this study combines conventional standard experiments and similarity simulation experiments to obtain the optimal three-dimensional flexible mesh parameters using mechanical characteristics as indicators, and it conducts numerical simulations to explore the support effect of three-dimensional flexible mesh on the tunnel inside the backfill under static loads in mines. Thereby, it comprehensively analyzes the enhancement effect of flexible mesh on the mechanical properties of the backfill material.

## 2. Experimental Materials and Methods

### 2.1. Experimental Materials

**Figure 1:**
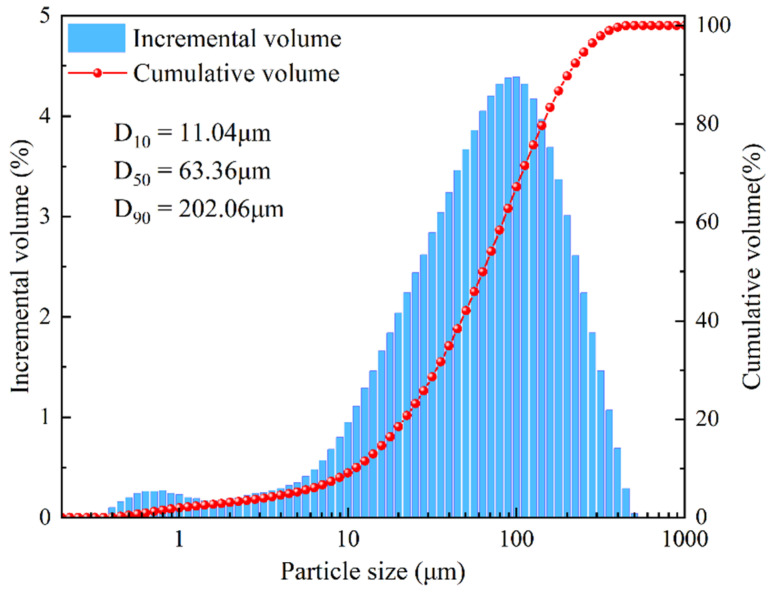
Particle size distribution of tailings.

The experimental tailings came from a certain iron mine in Anhui Province. The cementitious materials used were self-produced cemented powder in the mine and ordinary Portland cement made in Changsha, and the water used was tap water. The tailings were tested by a laser particle size analyzer (Mastersizer 2000 produced by Malvern), and the particle size distribution is shown in Figure 1. The physical parameters were tested according to the standard, and the results are shown in Table 1. The flexible mesh is made of polyethylene wire made in Changsha with a diameter of 0.7 mm and a tensile strength of 8 kg. Its single rope tensile strength is 40 MPa and the rope weight is 0.3 g/m. The molds used in the experiment include a 100 mm × 100 mm × 100 mm three-piece metal mold, a 400 mm × 400 mm × 400 mm acrylic-material mold box having an upper opening, and a 400 mm long, 120 mm × 120 mm cross-section polyethylene-material straight-wall arched-tunnel mold.

### 2.2. Experimental Methods

The experiment consisted of two parts. One part was a laboratory experiment with small samples, and the other part was a similarity simulation experiment with large samples. The purpose of the small-sample experiment was to determine the optimal spacing of the flexible mesh, and the purpose of the large-sample similarity simulation experiment was to compare and analyze the differences between backfill with flexible mesh and ordinary backfill without flexible mesh under external forces. The process for preparing the small samples was as follows: Make a slurry of tailings and cementitious materials with a cement-to-sand ratio of 1:4 and a mass concentration of 70%, and then inject the slurry into a three-piece metal mold. At the same time, place the three-dimensional flexible mesh with different spacings (30 mm, 40 mm, and 50 mm) into the metal mold to make backfill samples with the flexible mesh, and make control-group backfill samples without the flexible mesh. After the samples are formed, they should be placed in a curing box (temperature 20 ± 2 °C, humidity 95%) for curing. After curing for 28 days, uniaxial compressive strength, tensile strength, and shear strength tests should be conducted. The process for preparing large samples was as follows: Make a slurry of tailings and cement with a cement-to-sand ratio of 1:4 and a mass concentration of 70%, then inject the slurry into the mold box. At the same time, place the prepared tunnel mold and three-dimensional flexible mesh into the mold box to make similarity simulation samples. When placing the flexible mesh, it is necessary to place the flexible mesh on half of the reserved roadway but not on the other half, to form a comparative effect. After the sample is formed, it will be placed in a constant temperature and humidity environment with a temperature of 20 ± 2 °C and a humidity of 95% for curing. The test will be conducted after curing for 28 days.

**Figure 2:**
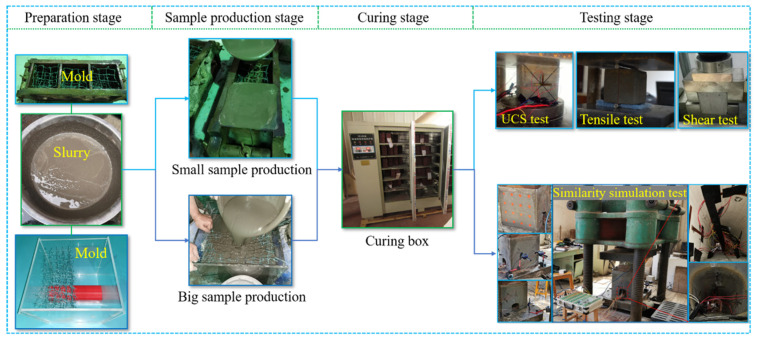
Experimental flow chart.

The compressive and tensile tests of the small specimens were conducted using a YNS600 electro-hydraulic servo universal testing machine made in China, with the displacement loading controlled at 3 mm/min. The shear test was conducted using an RJST-616 multifunctional shear test system. The similarity simulation test of the large samples was conducted using a YE-5000 hydraulic pressure testing machine made in Changchun. During the experiment, a JM3812 multifunctional static strain gauge made in China was used to record strain, and a high-definition camera was used to capture the development of cracks. The experimental process is shown in Figure 2.

## 3. Experimental Result

### 3.1. Mechanical Characteristics

**Figure 3:**
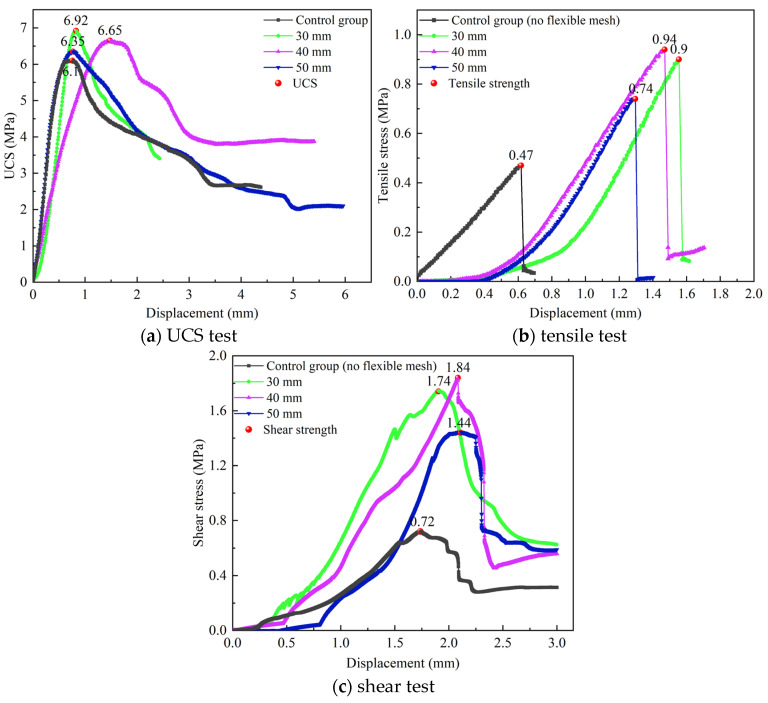
Stress-displacement diagram.

The laboratory test results of the small samples are shown in Figure 3. The results show that the stress-displacement curve trends of the backfill with flexible mesh and those of the backfill without flexible mesh are consistent. However, after adding the flexible mesh, the peak strength of the backfill increases, the peak stress point shifts backward, the ductility increases, and the brittleness decreases. Figure 3a shows that in the uniaxial compression test of the backfill material, it will go through a compaction stage, elastic stage, yield stage, and failure stage in sequence [19,20]. The peak strength was statistically analyzed, and the results are shown in Table 2. It can be seen from Table 2 that after adding the three-dimensional flexible mesh, the peak strengths (UCS, tensile strength, and shear strength) of the backfill have been improved; the tensile strength and shear strength, especially, have been greatly increased. The uniaxial compressive strength (UCS) of the backfill with the flexible mesh gradually decreases with the increase of spacing, but the decrease is not significant. For every 10 mm increase in spacing, the UCS decreases by about 5%, which indicates that reducing the spacing between flexible mesh helps to improve the UCS of the backfill material, but the increase effect is not significant. The tensile strength of the backfill with the flexible mesh increases first and then decreases as the mesh spacing decreases. There exists an optimal mesh spacing, which is 40 mm. The shear strength of the backfill with the flexible mesh increases first and then decreases with the decrease of mesh spacing. There exists an optimal mesh spacing, which is 40 mm.

### 3.2. Failure Mode Analysis

**Figure 4:**
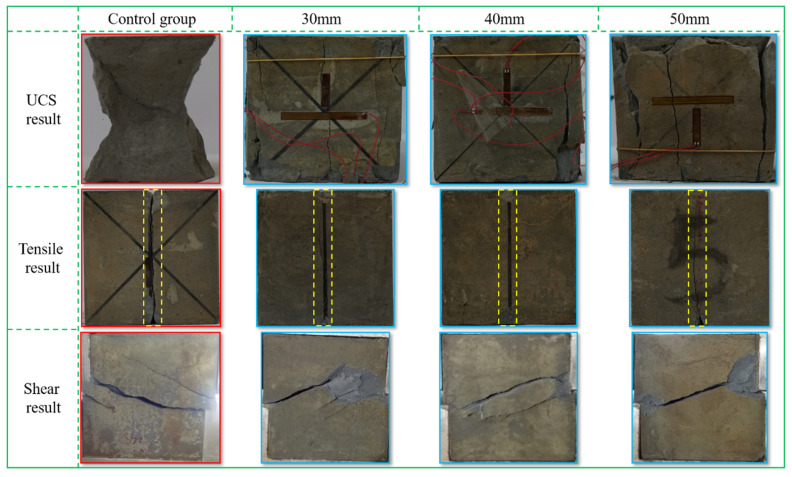
Failure modes of each test at different spacings.

The failure modes of the backfill with different mesh spacings are shown in Figure 4. From Figure 4, it can be seen that the failure modes obey a certain law. The results of the compression test indicate that the failure mode of the backfill is mainly conjugate with shear failure, ultimately forming a “dumbbell shape” having large ends and a small middle [21,22]. From the contour after destruction, it was found that the contour of the backfill sample with the flexible mesh remained relatively intact as a whole. The fragments of the edge caused by shear failure around the sample were relatively uniform in size, and most of them could autonomously adhere to the sample. There was no phenomenon of large pieces peeling off and falling off similar to the ordinary backfill sample (control group). The results of the tensile and shear tests indicate that the failure modes of the backfill were mainly tensile failure and shear slip failure. After the tensile and shear tests, the failure degree of the backfill with the flexible mesh was lower than that of the ordinary backfill sample. This is because, on the one hand, the shear strength of the flexible mesh is greater than the shear stress of the backfill, and the shear stress cannot cut the flexible mesh, and thus it is unable to form a large shear surface and unable to cause large-scale shear failure. On the other hand, adding a three-dimensional flexible mesh to the backfill changes the stress distribution in the backfill sample, reduces the local concentration of stress in the backfill, and indirectly improves the bearing strength of the backfill.

### 3.3. Strengthening Effect of Flexible Mesh

**Figure 5:**
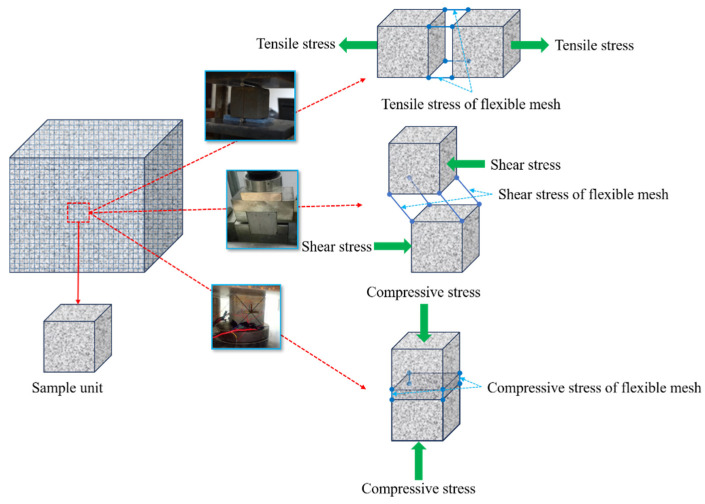
Schematic diagram of flexible mesh reinforcement mechanism.

In order to quantitatively evaluate the strengthening effect of adding flexible mesh to the backfill, a strengthening coefficient k is introduced, which is the ratio of the strength of the backfill with the flexible mesh to that without it. When *k* < 1, it indicates that the flexible mesh has a weakening effect on the mechanical properties of the backfill material; when *k* = 1, it indicates that the flexible mesh has no effect on the mechanical properties of the backfill material; when *k* > 1, it indicates that the flexible mesh has a strengthening effect on the mechanical properties of the backfill material. The calculation formula is as follows:

(1) $$k=\frac{P_{i}}{P_{0}}$$

where, *P_i_* represents the strength of the backfill material with different mesh spacings, and *P*_0_ represents the strength of the backfill material in the control group. The calculation results based on Equation (1) are shown in Table 3. It can be seen from Table 3 that the strengthening coefficients are all greater than 1, which indicates that the strengths have all increased. Among them, the UCS is 1.04–1.13 times that of the control group, the tensile strength is 1.57–2.00 times that of the control group, and the shear strength is 2.00–2.56 times that of the control group. This indicates that the mechanical properties of the backfill have been strengthened after adding the flexible mesh, and the strengthening mechanism is shown in Figure 5. When the flexible mesh is added to the backfill and combined with the backfill to form a whole, it is difficult to separate the backfill due to the traction effect of the flexible mesh during the tensile or shear tests. However, the compression test causes the flexible mesh to be compressed, making it difficult for it to function effectively. Therefore, the flexible mesh has a significant effect on the tensile and shear strengths of the backfill, but not as significant an effect on the compressive strength. From the strengthening coefficient, it can also be seen that the strengthening coefficient of the UCS is relatively small, while the strengthening coefficients of the tensile strength and shear strength are relatively large, which indicates that the addition of flexible mesh to the backfill significantly increases its tensile strength and shear strength, while the increase in the UCS is relatively small. Flexible mesh mainly enhances the tensile and shear strength of the backfill material, preventing its brittle failure, thereby increasing its toughness, ductility, and crack resistance to better meet engineering production needs.

### 3.4. Deformation and Failure of Backfill

**Figure 6:**
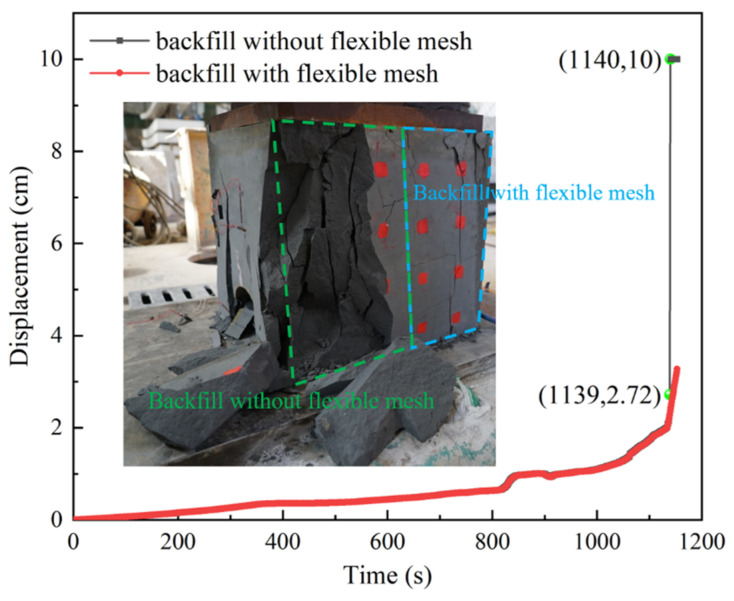
Surface displacement diagram.

The similarity simulation experimental results of the backfill with a reserved roadway are shown in Figure 6. From Figure 6, it can be observed that the surface displacement curves of the ordinary backfill and of the backfill with the flexible mesh exhibited similar trends in the initial phase but diverged in the later stage. At 1139 s, the surface displacement of the ordinary backfill changed suddenly, increasing from 2.72 cm to approximately 10 cm, indicating failure and spalling of the backfill, which led to a rapid surge in displacement. In contrast, the surface displacement of the backfill with the flexible mesh continued to increase at a relatively slow rate, indicating that the backfill maintained its structural integrity and exhibited strong load-bearing capacity.

### 3.5. Deformation and Failure of Surrounding Rock of Reserved Roadway in the Backfill

**Figure 7:**
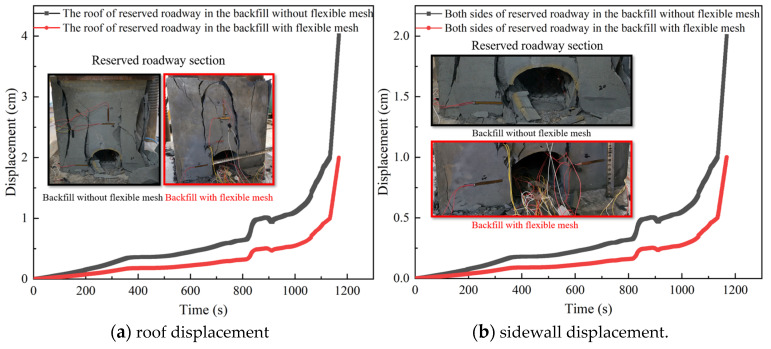
Surrounding rock displacement diagram of the reserved roadway in the backfill.

As shown in Figure 7a, during the testing process, the roof displacement trends of the reserved roadway in the backfill with the flexible mesh and the ordinary backfill without the flexible mesh exhibited similar displacement variation patterns but differed in magnitude. The roof displacement of the reserved roadway in the backfill with the flexible mesh was consistently lower than that of the ordinary backfill without the flexible mesh, with a nearly 50% reduction in maximum displacement during the later stages. This indicates that the roof stability of the reserved roadway in the backfill with the flexible mesh is superior to that of the ordinary backfill without the flexible mesh. As shown in Figure 7b, during the testing process, the sidewall displacement trend of the reserved roadway in the backfill with the flexible mesh and that of the ordinary backfill without the flexible mesh also followed similar patterns but differed in magnitude. The sidewall displacement of the reserved roadway in the backfill with the flexible mesh remained consistently lower than that of the ordinary backfill without the flexible mesh, with nearly a 50% reduction in maximum displacement during the later stages. This demonstrates that the sidewall stability of the reserved roadway in the backfill with the flexible mesh is superior to that of the ordinary backfill without the flexible mesh.

Due to the higher tensile and shear strengths of the backfill with the flexible mesh compared to the ordinary backfill without the flexible mesh, as well as its ability to retain structural integrity and significant load-bearing capacity even after failure, the roof and sidewalls of the reserved roadway in the backfill with the flexible mesh exhibit superior stability under identical loading conditions compared to those in the ordinary backfill without the flexible mesh.

## 4. Numerical Simulation

### 4.1. Model Building

**Figure 8:**
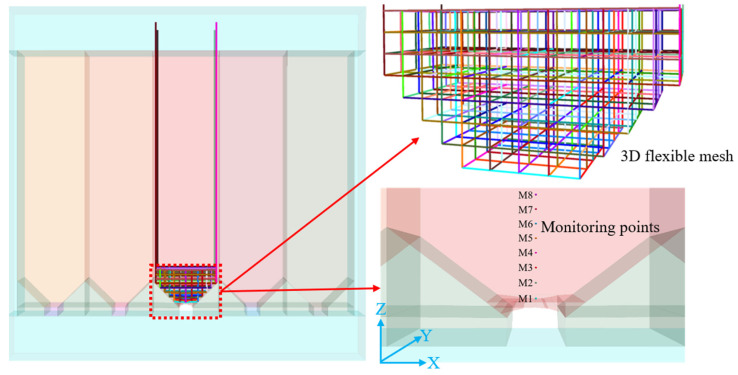
Numerical simulation model.

The numerical simulation was conducted using 3DEC 7.0 software. In the model (Figure 8), each stope has a width of 15 m and a length of 60 m, with a trench-type structure at the bottom for ore drawing. The stopes were mined using an alternate mining method of mining every other stope. The backfill includes two types: ordinary backfill and backfill with three-dimensional flexible mesh, where the flexible mesh is spaced at 1 m × 1 m × 1 m spacings, as shown in Figure 8.

The backfill zone was simulated using the Bonded Block Model (BBM). In the BBM region, the bulk can be elastic or elastoplastic, and the zone consists of block elements and interface elements. The interfaces between blocks were modeled using the Mohr–Coulomb contact criterion. The five surfaces of the model, namely the front, back, left, right and bottom, all adopt the constraint of fixed normal displacement. Based on experimental tests and field requirements, after parameter calibration, the mechanical parameters of the interfaces for backfill with a binder-to-sand ratio of 1:4 are listed in Table 4. The numerical model applied only self-weight stress, while an equivalent stress corresponding to the self-weight of a 600 m thick orebody (matching the actual mining depth of 600 m in the mine) was applied to the top of the model for static calculations. Eight monitoring points (M1–M8) were set above the roadway at 1 m intervals, as illustrated in Figure 8.

### 4.2. Simulation Result Analysis

#### 4.2.1. Displacement

**Figure 9:**
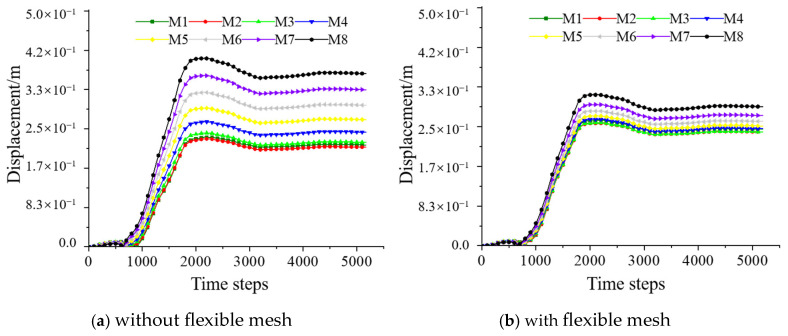
The total displacement of the monitoring points above the reserved tunneling.

The total displacements at each monitoring point of the reserved roadway in the backfill under static pressure are shown in Figure 9. From Figure 9, it can be observed that, in the absence of the three-dimensional flexible mesh, the final displacements at all the monitoring points reached an order of magnitude of 10^−1^ m, with a decreasing trend from bottom to top. Specifically, displacements were larger closer to the free surface of the roadway, and the influence range of the reserved roadway extended vertically to approximately 5 m. However, when the reserved roadway was reinforced with the three-dimensional flexible mesh, the displacements at all monitoring points converged toward a mean value. The displacements at the monitoring points near the roadway surface slightly increased, while the total displacements at the points beyond a vertical height of approximately 5 m decreased, with the maximum displacement reduced by 21.43%. This indicates that the three-dimensional flexible mesh reinforcement effectively constrained the overall deformation of the backfill above the roadway.

**Figure 10:**
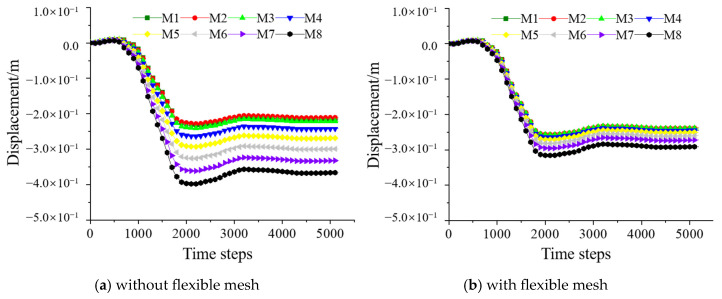
Displacements of the Z direction of the monitoring points above the reserved tunneling.

Since displacements in the X and Y directions were negligible, only the Z-direction displacement was analyzed (Figure 10). The variation in Z-direction displacement followed a pattern consistent with the total displacement, except that the Z-direction displacement was negative. Within the range of less than 3 m above the roadway, the displacements at the monitoring points with the flexible mesh were slightly larger compared to those without the mesh. However, in the range of 3–8 m above the roadway, the displacements with the flexible mesh were significantly smaller, reduced by over 20%. This further demonstrates that the three-dimensional flexible mesh reinforcement plays a role in constraining the support range of the reserved roadway in the backfill. Overall, an eight-layer three-dimensional flexible mesh effectively restricted roadway displacements.

#### 4.2.2. Strain Energy

**Figure 11:**
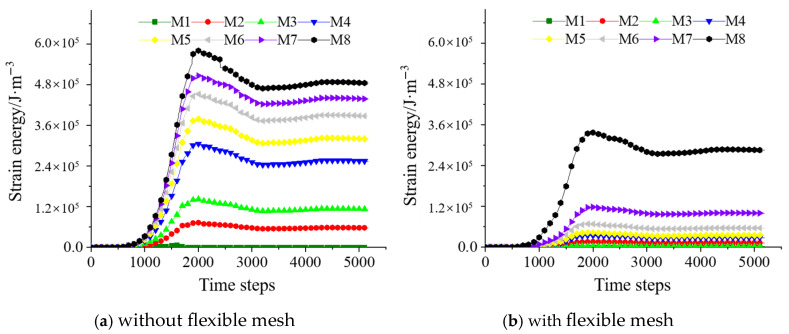
Strain energy of monitoring points above the reserved tunneling.

In practical engineering, stresses induce changes in the energy field of the backfill surrounding the roadway, indicating that the strain energy is closely related to the stress state of the backfill. The strain energy variations of backfill with and without three-dimensional flexible mesh at different vertical heights are shown in Figure 11.

As illustrated in Figure 11, the strain energy magnitude reaches the order of 10^5^ J/m^3^. In the absence of the three-dimensional flexible mesh, during the formation of the reserved roadway, the strain energy in the backfill increases from zero due to the combined effects of self-weight stress, overlying rock pressure, and lateral confinement forces from adjacent stopes. Near the roadway surface, the backfill experiences minimal compressive stress, resulting in relatively lower strain energy compared to other regions. As the vertical height increases, the strain energy of the backfill gradually rises, showing a positive correlation with height. When the three-dimensional flexible mesh is incorporated, the strain energy stored in the backfill is significantly reduced by approximately 40%. This reduction reflects a diminished stress state in the backfill, as part of the strain energy is transferred to the flexible mesh. Consequently, this mechanism effectively lowers the risk of failure in the reserved roadway within the backfill.

#### 4.2.3. Flexible Mesh State Analysis

**Figure 12:**
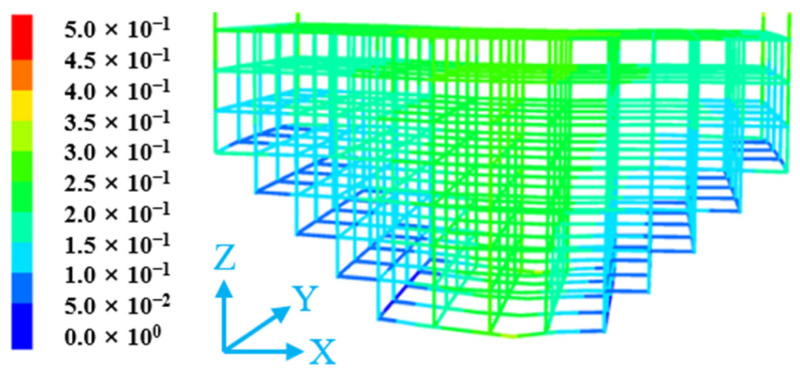
The displacement of the flexible mesh in the reserved tunneling.

Figure 12 shows the displacement of the three-dimensional flexible mesh in the reserved roadway under static stress. From Figure 12, it can be observed that the lower part of the flexible mesh experiences greater forces, with displacements exceeding 0.5 m. The structure undergoes significant displacement in its central region, reaching magnitudes in the order of 10^−1^ m, which radiate outward from the center toward both sides. In contrast, the displacements at the two lateral sides of the flexible mesh are relatively small.

**Figure 13:**
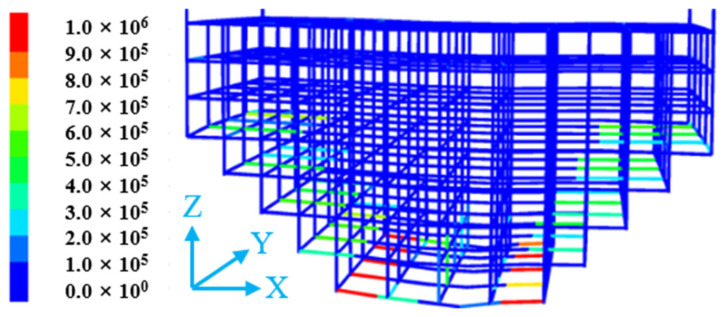
The tensile force of the flexible mesh in the reserved tunneling.

Figure 13 illustrates the tensile forces acting on the flexible mesh in the reserved roadway under static stress. The tensile force on the flexible mesh exhibits an inverse correlation with its displacement. The larger the displacement generated by the flexible mesh, the smaller the tensile force it experiences; conversely, the smaller the displacement, the greater the tensile force.

## 5. Conclusions

Flexible mesh has the characteristics of crack resistance and improved toughness, and it is not prone to agglomeration, thus having a good gain effect on the backfill material. This study investigated the influence of different mesh spacings on the mechanical properties of the backfill material, and it analyzed the support effect of flexible mesh on the reserved roadway in the backfill material under static pressure by similarity simulation experiments and numerical simulations. The main conclusions are as follows:

(1) The three-dimensional flexible mesh has a strengthening effect on the backfill. After adding the three-dimensional flexible mesh, the various strengths (UCS, tensile strength, and shear strength) of the backfill were improved, the tensile strength and shear strength, especially, being greatly increased. The UCS was 1.04–1.13 times that of the control group, the tensile strength was 1.57–2.00 times that of the control group, and the shear strength was 2.00–2.56 times that of the control group. The adhesion between the flexible mesh and the filling material was the main reason for the strength improvement.
(2) The three-dimensional flexible mesh can ensure the integrity of the backfill. After adding the three-dimensional flexible mesh, there was no fragment detachment of the backfill under external force, showing that it ensures the integrity of the backfill well. The adhesion between the flexible mesh and the fragments of the backfill makes it difficult for the fragments to fall off, which is the main way in which it ensures the integrity of the backfill.
(3) The three-dimensional flexible mesh can reduce the deformation of the surrounding rock in the reserved roadway. After adding the three-dimensional flexible mesh, the displacement trend of the roadway roof and two sides was similar to that without the three-dimensional flexible mesh, but the values were relatively small, and there was no sudden increase in displacement in the later stage. The maximum displacement in the later stage decreased by 50%.
(4) Under static pressure, the three-dimensional flexible mesh has a good supporting effect on the roadway roof. After adding the three-dimensional flexible mesh, the displacement and overall strain energy near the roadway roof in the backfill were smaller than those of the ordinary backfill without the flexible mesh, with a maximum reduction of 21.43% in displacement and a maximum reduction of 40% in strain energy.
(5) Although this study has undertaken much work and obtained many conclusions, there are still some aspects that need further research, such as the influences of dynamic and cyclic loads.

## Figures and Tables

**Table 1:** Physical parameters of tailings.

Classification	Density (kg/m^3^)	Max Porosity (%)	Min Porosity (%)	Natural Repose Angle (°)
value	1943	49.5	37.5	37.6

**Table 2:** Strength of flexible mesh backfill at different spacings.

Group	Control Group	30 mm	40 mm	50 mm
UCS/MPa	6.10	6.92	6.65	6.35
tensile strength/MPa	0.47	0.90	0.94	0.74
shear strength/MPa	0.72	1.74	1.84	1.44

**Table 3:** Strengthening coefficients of flexible mesh backfill at different spacings.

Group	30 mm	40 mm	50 mm
UCS/MPa	1.13	1.09	1.04
tensile strength/MPa	1.91	2.00	1.57
shear strength/MPa	2.42	2.56	2.00

**Table 4:** Mechanical parameters of the contact surface after BBM area correction.

Normal Stiffness (GPa/m)	Stiffness (GPa/m)	Compressive Strength (MPa)	Tensile Strength (MPa)	Internal Friction Angle (°)	Cohesion (MPa)
0.3	0.3	2.116	0.181	36.94	0.171

## Data Availability

The original contributions presented in this study are included in the article. Further inquiries can be directed to the corresponding author.
